# Systematic Review of the Socioeconomic Consequences in Patients With Multiple Sclerosis With Different Levels of Disability and Cognitive Function

**DOI:** 10.3389/fneur.2021.737211

**Published:** 2022-01-06

**Authors:** Andrius Kavaliunas, Virginija Danylaitė Karrenbauer, Stefanie Binzer, Jan Hillert

**Affiliations:** ^1^Department of Clinical Neuroscience, Karolinska Institutet, Stockholm, Sweden; ^2^Neurology Medical Unit, Karolinska University Hospital, Huddinge, Sweden; ^3^Department of Neurology, Kolding Hospital, Kolding, Denmark

**Keywords:** multiple sclerosis (MS), employment, socioeconomic factors, income, sick leave, systematic review, disability evaluation, cognition

## Abstract

Multiple sclerosis (MS) is a challenging and disabling condition, predominantly affecting individuals in early adulthood. MS affects the physical, cognitive, and mental health of persons suffering from the disease as well as having a great impact on their financial status and quality of life. However, there is a lack of systematic approach toward assessing the socioeconomic consequences of MS. Our objective was to systematically review analytical observational studies investigating the socioeconomic consequences in persons with MS with different levels of physical disability and cognitive function. We conducted a systematic review on socioeconomic consequences of MS with a focus on employment-, income-, work ability-, and relationship-related outcomes in persons with MS with special focus on disability and cognition. Additionally, the educational characteristics were examined. From 4,957 studies identified, 214 were assessed for eligibility and a total of 19 studies were included in this qualitative assessment; 21 different outcomes were identified. All identified studies reported higher unemployment, higher early retirement, and higher risk of unemployment in relation to higher physical disability. Also, cognitive function was found to be a predictor of employment (unemployment). The studies pointed out significant correlations between greater disability and lower earnings and higher income from benefits. A study found the same correlation in relation to cognitive function. The studies reported higher work disability in relation to higher physical disability and lower cognitive function. In conclusion, this systematic review summarizes the pronounced differences in various socioeconomic outcomes between patients with MS with regards to their physical disability and cognitive function. In addition, we identified a lack of studies with longitudinal design in this field that can provide more robust estimates with covariate adjustments, such as disease modifying treatments.

## Introduction

Approximately 2.8 million people worldwide are affected with multiple sclerosis (MS)—a chronic demyelinating and neurodegenerative disease of the central nervous system with increasing prevalence (1, 2). MS poses a major personal and socioeconomic burden: the average age of disease onset is 30 years—a time that is decisive for work and family planning; persons with MS die 7–10 years earlier and live on average almost 20 years with moderate and 30 years with severe disability (1, 3, 4). The condition has a heterogeneous presentation that can include sensory and visual disturbances, motor function impairments, fatigue, pain, and cognitive deficits (1, 5).

Within the MS population, the spectrum of disability ranges from essentially unaffected to highly disabled. The most common measure utilized to assess physical disability is the Expanded Disability Status Scale (EDSS) (6), which is based on a standardized neurological examination in combination with assessment of walking distance, arm function, speech, and utilization of walking aid and wheelchair; however, it is non-linear and some functional score items are evaluated subjectively. As the disability of patients increases, they become dependent on their family for carrying out their daily routines and activities, which leads to a reduction of their quality of life (7). Even in a population with low physical disability, MS is responsible for a substantial economic burden due to indirect and informal care costs (8). In addition, we previously reported that the average level of earnings was ten times lower and the average level of health-related benefits was four times higher when comparing patients with MS with severe and mild disability (9).

Cognitive decline is recognized as a prevalent and debilitating symptom of multiple sclerosis (10). Measurable cognitive dysfunction has been reported in up to 70% of patients (11). Various aspects of cognitive function can be detrimentally affected: difficulties with long-term and verbal memory, abstract and conceptual reasoning, fluency, planning, visuospatial perception, and reduced speed of information processing (11). In addition, the cognitive function affects the financial situation of persons with MS negatively, independently of physical disability, e.g., persons with MS in the highest Symbol Digit Modalities Test (SDMT) quartile earned more than two times annually compared with those in the lowest SDMT quartile (12).

Previously, we summarized the pronounced differences between patients with MS and the general population, e.g., 15–30% lower employment, lower earnings and higher social benefits, higher absenteeism and presenteeism proportions, and higher work disability (e.g., sick leave days) among persons with MS (13). However, besides underlying differences between MS and general population, persons with MS are quite different in terms of progression of physical disability, reduction of cognitive function, etc. As socioeconomic outcomes can be investigated in many ways (e.g., income, employment, marital status, sick leave days, etc.) a comprehensive overview is warranted. Thus, our aim was to systematically review the studies investigating the socioeconomic consequences in persons with MS in regard to their physical disability and cognition.

## Methods

We conducted a systematic review following the Preferred Reporting Items for Systematic Reviews and Meta-Analyses (PRISMA) statement (14). The study protocol was registered in PROSPERO: International prospective register of systematic reviews (https://www.crd.york.ac.uk/prospero/), ID: CRD42020182085. Published studies on socioeconomic consequences of MS were systematically searched in Medline (Ovid), Embase, and Web of Science (Clarivate). A combination of relevant keywords to construct the search strategy, such as MS, socioeconomic outcomes, employment, income, earnings, benefits, disability pension, sickness absence, sick leave, and marital status, was used (full search strategy is available in Supplementary Material 1). The search was limited to English language and publications prior to July 2019.

One author (AK) conducted the first screening of potentially relevant records based on titles and abstracts, and two authors (AK and VDK) independently performed the final selection of included studies based on full text evaluation against the eligibility criteria. Rayyan, a web and mobile app for systematic reviews (https://rayyan.qcri.org/welcome) was used to facilitate the review process. Consensus between the two reviewers was used to resolve any disagreement.

The main eligibility criteria were:

- Population: adults of working age;
- Exposures: higher physical disability level assessed by EDSS (6); lower cognitive function assessed by SDMT (15);
- Comparators: lower physical disability level assessed by EDSS (6); higher cognitive function assessed by SDMT (15);
- Outcomes: socioeconomic outcomes (employment, income, work ability, education, and relationship);
- Study design: analytical observational studies (e.g., cohort, case-control, and cross-sectional) (16), excluding descriptive studies, case reports, and case series. Clinical trials and economic evaluations (e.g., cost if illness and cost-effectiveness studies) were not in the scope of this review.

**Figure 1:**
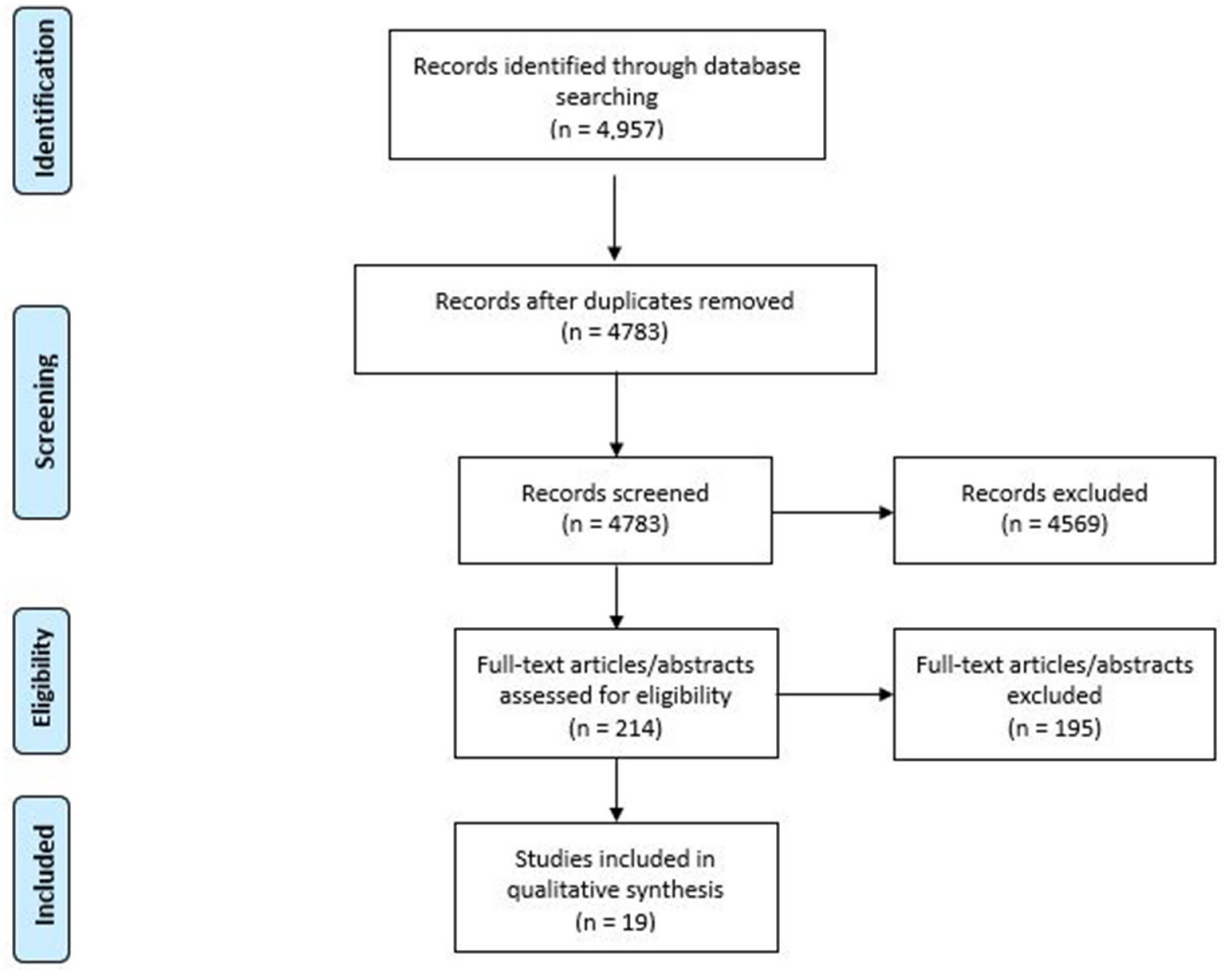
PRISMA flow diagram.

Initially 4,957 studies were identified (Figure 1) and 4,783 records were screened after duplicates were removed. In total, 214 full-text articles (or abstracts) were assessed for eligibility and finally 19 studies were included in the qualitative synthesis. Only full articles were considered for the qualitative analysis. Studies that did not report any estimates (e.g., proportions and ratios), only pointing out to the direction (higher, lower) or association (e.g., significant and not significant) were not considered for the evaluation. Using a standardized data extraction form in Excel, study characteristics (as presented in the tables and Supplementary Material 2) were extracted from the included studies. In case of possible overlap in a study population, the most recent study was selected.

Three reviewers (AK, VDK, and SB; two per each study) independently assessed the quality of the included studies focusing on such study characteristics as study design (cohort studies prioritized over cross-sectional), data sources (registries and databases prioritized over surveys and interviews), timeline, and the size of the study population. Accordingly, on the basis of the aforementioned study characteristics, each study was evaluated by points, whereby this is reflected in the final grading (A—high quality; B—moderate quality; C—low quality). Consensus was used to resolve any disagreement. The quality assessment was performed ensuring that at least one of the reviewers is not among the co-authors (Supplementary Material 2).

Our aim was to broadly review the studies with regards to the socioeconomic outcomes analyzed, thus we applied a comprehensive search strategy to include all possible operationalized definitions. In total, 21 different outcomes were identified. We grouped the outcomes into the categories of employment-, income-, work ability-, and relationship-related outcomes, also mapping the reported indicators for each of the included study (Table 1). Additionally, we extracted the information about the educational level from the selected studies. The employment domain consisted of such keywords as employment, unemployment, work, labor, occupational status; income domain—income, salary, earnings, benefits, compensation, remuneration; the work ability domain—work ability, work disability, sick leave, sickness absence, disability pension, absenteeism, presenteeism; relationship domain: marital status, relationship status, divorce, etc. (Supplementary Material 1). Results from the studies were qualitatively compared and summarized. We used the original categorization of EDSS and SDMT values, as it was reported in the studies. As many authors use different categories, we classified the lowest reported EDSS category into “lower EDSS” and the remaining into “higher EDSS,” similarly for SDMT.

**Table 1:** The list of socioeconomic outcomes.

**Outcomes**	**References**
**Employment-related outcomes:**	
Proportion of the employed (fully or partially) or unemployed;	(17–25)
Proportion of early retirement;	(19, 22, 24)
Odds ratio (OR) for employment (unemployment).	(18, 20, 23, 26–28)
**Income-related outcomes:**	
Mean annual income (earnings, benefits);	(9, 12)
Median annual income (earnings, benefits);	(12, 18)
Proportion receiving earnings (earnings >0);	(9, 12)
Proportion receiving social benefits;	(9, 12, 19, 24)
Percental difference in income;	(9)
OR for having income (earnings, benefits);	(9, 12)
Prevalence ratio for having income (earnings, benefits);	(9, 12)
Adjusted regression coefficient for amount of income (earnings, benefits).	(9, 12)
**Work ability-related outcomes:**	
OR for full and/or partial sick leave;	(29)
Proportion on full-time disability pension;	(30)
Absenteeism (correlation coefficient, regression coefficient);	(31)
Presenteeism (correlation coefficient, regression coefficient);	(31)
Proportion on absence at work;	(32)
Work disability (annual net days of sickness absence and disability pension);	(33)
Incidence rate ratio (IRR) for work disability;	(33)
Predicted marginal mean of work disability.	(33)
**Relationship outcomes:**	
Proportion of a relationship status (e.g., married/cohabitant, single).	(9, 33)
**Educational level:**	
Proportion of those having school/high-school/university education.	(9, 12, 33)

## Results

A total of 19 studies were selected for inclusion into this systematic review of socioeconomic consequences of MS in relation to physical disability and cognitive function. Of them, 18 studies provided data from their respective countries, and one study was multi-center with the results from 16 European countries. The majority of studies (12 of 17) were conducted in Europe (with half of them in the Scandinavian countries, i.e., five in Sweden and one in Norway); four studies—in North America (three in USA and one in Canada); one-study in Hong Kong and one in New Zealand.

With respect to study design, three were cohort studies and 16—cross-sectional studies; four studies analyzed data from the registries, whereas 14 studies included data from surveys, questionnaires, or interviews (note, one study did not provided information about the data source used). The selected studies are summarized in the Tables 2–9 and categorized according to the functional domain (physical disability or cognitive function), and type of outcome.

**Table 2:** Studies that investigated employment-related outcomes in patients with multiple sclerosis (MS) within different levels of physical disability.

**First author**	**Country**	**Population (n)**	**Main results**		
			**Outcomes**	**Lower EDSS**	**Higher EDSS**
Battaglia et al. (19)	Italy	1010	Employed or self employed	0–3: **68.6%**	4–6.5: **45.4%**; 7–9: **16.0%**
			Working full-time	0–3: **3.0%**	4–6.5: **7%**; 7–9: **3.8%**
			Early retired	0–3: **0.6%**	4–6.5: **5.5%;** 7–9: **14.8%**
Boe Lunde et al. (20)	Norway	213	Employed	0–3: **70.8%**	3.5–6: **39.6%**; >6: **6.1%**
			OR (95% CI) for employment	0–3: 1	3.5–6: **0.27** (0.14–0.52); >6: **0.027** (0.06–0.12)
			Adjusted OR (95% CI) for unemployment	0–3: 1	3.5–6: **0.34** (0.15–0.77); >6: **0.05** (0.01–0.26)
Busche et al. (21)	Canada	96	Employed	0–2.5: **60.0%**	3–5.5: **28%;** 6–8: **12%;** 8.5–9.5%: **0%**
Findling et al. (22)	Switzerland	405	Full-time working	0–2.5: **41.4%**	3–4.5: **21.5%;** >5: **4.9%**
			Part-time working due to MS	0–2.5: **20.6%**	3–4.5: **35.6%**; >5: **18.5%**
			Full-time retired due to MS	0–2.5: **3.4%**	3–4.5: **32**.2%; >5: **69.2%**
Kobelt et al. (17)	16 countries^*^	16,808	Workforce participation: proportion of patients below retirement age employed or self-employed	0: **82%** 1: **77%** 2: **68%** 3: **54%** 4: **49%**	5: **39%** 6: **29%;** 6.5: **28%** 7: **16%** 8: **15%** 9: **8%**
Koziarska et al. (26)	Poland	150	OR (95% CI) for unemployment	0–3: 1	>3: **13.227** (5.221–38.741)
			Adjusted OR (95% CI) for unemployment	0–3: 1	>3: **11.089** (4.116–34.201)
Lau et al. (23)	Hong Kong	59	Employed	≤ 5.5: **97%**	>5.5: **3%**
			OR (95% CI) for employment	≤ 5.5: 1	>5.5: **0.071** (0.003–1.775)
MacLurg et al. (24)	UK	149	Employed	0–4.5: **43%**	5–6.5: **21%**; 7–9.5: **8%**
			Medically retired	0–4.5: **34%**	5–6.5: **51%**; 7–9.5: **75%**
Pearson et al. (18)	New Zealand	1727	Proportion not working	<3: **30.5%**	3–6: **50.6%** >6: **84.8%**
			OR (95% CI) for not working	<3: 1	3–6: **2.3** (1.8–2.9) >6: **12.7** (9.3–17.5)
			Adjusted OR (95% CI) for unemployment	<3: 1	3–6: **2.05** (1.59–2.64) >6: **9.32** (6.66–13.19)

*EDSS, Expanded Disability Status Scale; OR, odds ratio*.

* *Austria, Belgium, Czech Republic, Denmark, France, Germany, Hungary, Italy, the Netherlands, Poland, Portugal, Russia, Spain, Sweden, Switzerland, the United Kingdom. Bold values shows the estimate*.

**Table 3:** Studies that investigated employment-related outcomes in patients with MS within different levels of cognitive function.

**First author**	**Country**	**Population**	**Main results**		
			**Outcomes**	**Higher SDMT**	**Lower SDMT**
Campbell et al. (25)	UK	62	Employment rate	60–80: **100%** 50–60: **~60%**	40–50: **~45%** 30–40: **~20%** 20–30: **~0%**
Fraser et al. (28)	US	95	OR for fully employed vs. unemployed for a 1 SD difference in measure	SDMT written – 1.76 (0.89–3.53) SDMT oral – 1.46 (0.75–2.83)	
Morrow et al. (27)	US	97	Adjusted OR was 4.2 (95% CI, 1.2–14.8) of a deterioration in employment (paid disability benefits or a reduction in working hours) based on a change of SDMT by 4.0		

*CI, confidence intervals; OR, odds ratio; SDMT, Symbol Digit Modalities Test. Bold values shows the estimate*.

**Table 4:** Studies that investigated income-related outcomes in patients with MS within different levels of physical disability.

**First author**	**Country**	**Population (n)**	**Main results**		
			**Outcomes**	**Lower EDSS**	**Higher EDSS**
Battaglia et al. (19)	Italy	1010	Invalidity pension	0–3: **1.7%**	4–6.5: **19.2%**; 7–9: **39.1%**
Kavaliunas et al. (9)	Sweden	7929	Earnings >0	0–3.5: **84.7%**	4–5.5: **57.9%**; 6–6.5: **44.0%**; 7–9.5: **21.0%**
			Benefits >0	0–3.5: **52.3%**	4–5.5: **85.0%**; 6–6.5: **95.4%**; 7–9.5: **99.4%**
			Earnings, mean in SEK 100	0–3.5: **2140.9**	4–5.5: **1154.5**; 6–6.5: **763.6**; 7–9.5: **218.7**
			Health related benefits, mean in SEK 100	0–3.5: **349.5**	4–5.5: **932.1**; 6–6.5: **1232.2**; 7–9.5: **1419.8**
			Disability pension	241.3	720.3; 1019.6; 1239.4
			Sickness absence	102.9	177.2; 136.3; 53.7
			Disability allowance	5.3	34.7; 76.3; 126.8
			Benefits related to low income, mean SEK 100	0–3.5: **36.0**	4–5.5: **22.7**; 6–6.5: **8.9**; 7–9.5: **15.2**
			Unemployment compensation	29.5	14.6; 4.2; 3.1
			Social assistance	6.6	8.1; 4.7; 12.1
			Percentage change in earnings	0–3.5: reference	4–5.5: **−21.1%**; 6–6.5: **−31.3%**; 7–9.5: **−58.8%**
			Percentage change in benefits	0–3.5: reference	4–5.5: **+49.3%**; 6–6.5: **+73.0%**; 7–9.5: **+92.0%**
			Adjusted OR (95% CI) for having earnings^*^	0–3.5: 1	4–5.5: **0.32** (0.27–0.37); 6–6.5: **0.21** (0.17–0.24); 7–9.5: **0.07** (0.06–0.09)
			Adjusted OR (95% CI) for having benefits^*^	0–3.5: **1**	4–5.5: **4.06** (3.33–4.96); 6–6.5: **12.72** (9.09–17.80); 7–9.5: **89.13** (36.73–216.28)
			Adjusted RR (95%) for having earnings	0–3.5: **1**	4–5.5: **0.75** (0.71–0.79); 6–6.5: **0.63** (0.57–0.67); 7–9.5: **0.33** (0.29–0.39)
			Adjusted RR (95%) for having benefits	0–3.5: **1**	4–5.5: **1.58** (1.52–1.64); 6–6.5: **1.81** (1.76–1.85); 7–9.5: **1.93** (1.90–1.94)
			Adjusted regression (95% CI) coefficient for level of earnings	0–3.5: reference	4–5.5: **−658.11** (**–**816.21– **–**500.02); 6–6.5: **−945.04** (**–**1133.71– **–**756.36); 7–9.5: **−1669.31** (**–**1939.91– **–**1398.72)
			Adjusted regression (95% CI) coefficient for level of benefits	0–3.5: reference	4–5.5: **285.50** (244.92–326.08); 6–6.5: **422.74** (381.30–464.18); 7–9.5: **545.34** (501.90–588.78)
MacLurg et al. (24)	UK	149	Disability related income Other benefits	0–4.5: **40%** 0–4.5: **6%**	5–6.5: **89%**; 7–9.5: **91%** 5–6.5: **23%**; 7–9.5: **15%**
Pearson et al. (18)	New Zealand	1727	Median income (NZD)	<3: **30.000**	3–6: **20.000**; >6: **15.000**

*EDSS, Expanded Disability Status Scale; OR, odds ratio; RR, risk ratio*.

* *Crude ORs are available in the original article but were not extracted to this review. Bold values shows the estimate*.

**Table 5:** Studies that investigated income-related outcomes in patients with MS within different levels of cognitive function.

**First author**	**Country**	**Population**	**Main results**		
			**Outcomes**	**Higher SDMT**	**Lower SDMT**
Kavaliunas et al. (12)	Sweden	2080	Proportion of earnings >0	QIV (62–110): **91.1%** QIII (54–61): **85.6%**	QII (45–53): **80.6%** QI (6–44): **59.2%**
			Earnings mean (SEK 100)	QIV (62–110): **2282** QIII (54–61): **1968**	QII (45–53): **1728** QI (6–44): **1046**
			Earnings median (SEK 100)	QIV (62–110): **2183** QIII (54–61): **1841**	QII (45–53): **1537** QI (6–44): **239**
			Proportion of benefits >0	QIV (62–110): **48.5%** QIII (54–61): **62.1%**	QII (45–53): **64.5%** QI (6–44): **83.5%**
			Benefits mean (SEK 100)	QIV (62–110): **287** QIII (54–61): **495**	QII (45–53): **586** QI (6–44): **898**
			Benefits median (SEK 100)	QIV (62–110): **0** QIII (54–61): **161**	QII (45–53): **393** QI (6–44): **966**
			Adjusted OR (95% CI) for having earnings	QIV: **3.36** (2.23–5.07) QIII: **2.40** (1.69–3.41)	QII: **1.96** (1.44–2.66) QI: 1
			Corrected PR (95% CI) for having earnings	QIV: **1.40** (1.29–1.49) QIII: **1.31** (1.20–1.41)	QII: **1.25** (1.14–1.34) QI: 1
			Adjusted OR (95% CI) for having benefits	QIV: **0.41** (0.29–0.59) QIII: **0.57** (0.40–0.80)	QII: **0.51** (0.36–0.71) QI: 1
			Corrected PR (95% CI) for having benefits	QIV: **0.81** (0.71–0.90) QIII: **0.89** (0.80–0.96)	QII: **0.86** (0.78–0.94) QI: 1
			Adjusted coefficient (95% CI) for amount of earnings (estimate in SEK 100)	QIV: **722** (504–941) QIII: **497** (288–707)	QII: **403** (200–606) QI: Reference
			Adjusted coefficient (95% CI) for amount of benefits (estimate in SEK 100)	QIV: **−210** (−296–−123) QIII: **−93** (−170–−53)	QII: **−58** (−128–13) QI: Reference

*OR, odds ratio; PR, prevalence ratio; Q, quartile; SDMT, Symbol Digit Modalities Test. Bold values shows the estimate*.

**Table 6:** Studies that investigated work ability-related outcomes in patients with MS within different levels of physical disability.

**First author**	**Country**	**Population (n)**	**Main results**		
			**Outcomes**	**Lower EDSS**	**Higher EDSS**
Doesburg et al. (32)	The Netherlands	90	Low work absence (<1 month)	0–3.5: **81.6%**	≥4: **14.3%**
			High work absence (≥1 month)	0.3–5: **68.3%**	≥4: **31.7%**
Glanz et al. (31)	US	377	Spearman correlation coefficient (95% CI) between EDSS and absenteeism	0.09 (**–**0.04–0.21)
			Regression coefficient (95% CI) for absenteeism (adjusted)	0.38 (**–**1.12–2.39)
			Spearman correlation (95% CI) between EDSS and presenteeism	0.33 (0.21–0.43)
			Regression coefficient (95% CI) for presenteeism (adjusted)	3.60 (1.7–6.6)
Kavaliunas et al. (33)	Sweden	903	Adjusted IRR (95% CI) for work disability after 1 year	0–3.5: 1	4–5.5: **1.78** (1.49.2.12) 6–6.5: **2.08** (1.69–2.55) 7–9.5: **2.42** (1.72–3.39)
			Adjusted IRR (95% CI) for work disability after 3 years	0–3.5: 1	4–5.5: **1.88** (1.59–2.22) 6–6.5: **2.23** (1.84–2.70) 7–9.5: **2.61** (1.90–3.60)
Sundström et al. (29)	Sweden	399	Crude OR (95% CI) for full sick leave	0–2–5: 1	3–5.5: **4.5** (2.4–8.5) >6: **42** (19–95)
			Adjusted OR (95% CI) for full sick leave	0–2–5: 1	3–5.5: **3.5** (1.6–7.5) >6: **34** (13–86)
			Crude OR (95% CI) for partial or full sick leave	0–2–5: 1	3–5.5: **7.4** (3.9–14) >6: **166** (22–1200)
			Adjusted OR (95% CI) for full sick leave	0–2–5: 1	3–5.5: **6.5** (3.0–14) >6: **150** (19–1200)

*EDSS, Expanded Disability Status Scale; IRR, incidence rate ratio; OR, odds ratio. Bold values shows the estimate*.

**Table 7:** Studies that investigated work ability-related outcomes in patients with MS within different levels of cognitive function.

**First author**	**Country**	**Population**	**Main results**		
			**Outcomes**	**Higher SDMT**	**Lower SDMT**
Chruzander et al. (30)	Sweden	114	Proportion not on full-time disability pension	No impairment: **57%**	Impaired cognitive function: **43%**
Glanz et al. (31)	US	377	Pearson correlation coefficient (95% CI) between SDMT and absenteeism	**–**0.08 (**–**0.18– **–**0.002)	
			Regression coefficient (95% CI) for absenteeism (adjusted)	**–**0.09 (**–**0.25–0.02)	
			Pearson correlation (95% CI) between SDMT and presenteeism	0.08 (**–**0.18–0.03)	
			Regression coefficient (95% CI) for presenteeism (adjusted)	0.06 (**–**0.08–0.20)	
Kavaliunas et al. (33)	Sweden	903	Work disability at baseline	QIV: **98.5** QIII: **141.2**	QII: **182.2** QI: **229.9**
			Adjusted IRR (95% CI) for disability after 1 year	QIV: 1 QIII: **1.33** (1.111.60)	QII: **1.41** (1.18–1.70) QI: **1.73** (1.42–2.10)
			Adjusted IRR (95% CI) for disability after 3 years	QIV: 1 QIII: **1.22** (1.03–1.45)	QII: **1.33** (1.12–1.58) QI: **1.68** (1.40–2.02)
			Predicted marginal mean of work disability (annual days) after 1 year	QIV: **143** QIII: **191**	QII: **203** QI: **247**
			Predicted marginal mean of work disability (annual days) after 3 years	QIV: **154** QIII: **188**	QII: **206** QI: **259**

*IRR, Incidence rate ratio; Q, quartile; SDMT, Symbol Digit Modalities Test. Bold values shows the estimate*.

**Table 8:** Studies that investigated relationship outcomes and educational level in patients with MS within different levels of physical disability.

**First author**	**Country**	**Population (n)**	**Main results**		
			**Outcomes**	**Lower EDSS**	**Higher EDSS**
***Relationship outcomes***
Kavaliunas et al. (9)	Sweden	7929	Family composition:		
			Living with partner, no children	0–3.5: 13.6%	4–5.5: 24.5%; 6–6.5: 30.2%; 7–9.5: 24.1%
			Living with partner and with children	0–3.5: 44.1%	4–5.5: 33.9%; 6–6.5: 26.8; 7–9.5: 17.3%
			Single, no children	0–3.5: 33.6%	4–5.5: 32.5%; 6–6.5: 35.5%; 7–9.5: 52.7%
			Single, with children	0–3.5: 8.8%	4–5.5: 9.2%; 6–6.5: 7.5%; 7–9.5: 5.9%
***Educational level***
Kavaliunas et al. (9)	Sweden	7929	Lower	0–3.5: 8.2%	4–5.5: 14.5%; 6–6.5: 17.6%; 7–9.5: 18.6%
			Secondary	0–3.5: 45.3%	4–5.5: 50.7%; 6–6.5: 49.3%; 7–9.5: 49.8%
			Higher	0–3.5: 46.5%	4–5.5: 34.8%; 6–6.5: 33.1%; 7–9.5: 31.6%

**Table 9:** Studies that investigated relationship outcomes and educational level in patients with MS within different levels of cognitive function.

**First author**	**Country**	**Population**	**Main results**		
			**Outcomes**	**Higher SDMT**	**Lower SDMT**
***Relationship outcomes***		
Kavaliunas et al. (33)	Sweden	903	Married/cohabitating	QIV (57–86): **52.7%** QIII (49–56): **49.5%**	QII (40–48): **55.2%** QI (0–39): **47.2%**
			Single	QIV (57–86): **47.3%** QIII (49–56): **50.5%**	QII (40–48): **44.8%** QI (0–39): **52.8%**
***Educational level***		
Kavaliunas et al. (12)	Sweden	2080	Lower	QIV (62–110): **2.5%** QIII (54–61): **7.3%**	QII (45–53): **9.0%** QI (6–44): **15.8%**
			Secondary	QIV (62–110): **40.8%** QIII (54–61): **41.7%**	QII (45–53): **51.3%** QI (6–44): **52.9%**
			Higher	QIV (62–110): **56.7%** QIII (54–61): **51.0%**	QII (45–53): **39.7%** QI (6–44): **31.4%**
Kavaliunas et al. (33)	Sweden	903	Lower and secondary	QIV (57–86): **42.4%** QIII (49–56): **52.8%**	QII (40–48): **63.8%** QI (0–39): **65.2%**
			Higher	QIV (57–86): **57.6%** QIII (49–56): **47.2%**	QII (40–48): **36.2%** QI (0–39): **34.8%**

*Q, quartile; SDMT, Symbol Digit Modalities Test. Bold values shows the estimate*.

### Employment-Related Outcomes

We identified 12 studies that investigated the employment-related outcomes: nine in relation to physical disability (Table 2) and three in relation to cognitive function (Table 3). The largest study conducted in 16 European countries, which included 16,808 patients with MS, found gradually decreasing workforce participation (proportion of the patients below retirement age employed or self-employed) in relation to physical disability from 82% at EDSS 0 to 8% at EDSS 9 (17). Similarly, a study in New Zealand (18) that surveyed 1,727 patients with MS showed much higher proportion of persons not working with increasing EDSS levels: 84.8% among those with EDSS >6, 50.6% among those with EDSS 3–6, and 30.5% among those with EDSS <3. Correspondingly, a study in Italy (19) that included 1,010 patients with MS reported similar findings: the proportion of employed patients with MS was 16% at EDSS 7–9, 45.4% at EDSS 4–6.5, and 68.6% at EDSS 0–3. Other smaller studies in Norway (20), Canada (21), Switzerland (22), Hong Kong (23), and the United Kingdom (24) presented similar results.

In addition, three studies explored early retirement due to disease (medical retirement, and invalidity) pointing to a higher proportion among those with higher physical disability level, e.g., 75% among those with EDSS 7–9.5 in the United Kingdom (24), and 69.2% among those with EDSS >5 in Switzerland, (22) but 14.8% among those with EDSS 7–9 in Italy (19).

Four studies investigated and reported odds ratios (*OR*s) for employment/unemployment. With regards to unemployment, in New Zealand adjusted *OR* for unemployment was 2.05 (95% *CI*, 1.59–2.64) for those with EDSS 3–6, and 9.32 (95% *CI*, 6.66–13.19) for those with EDSS > 6, when compared with those with EDSS <3 (18). This was more pronounced in Poland with adjusted *OR* for unemployment of 11.089 (95% *CI*, 4.116–34.201) for those with EDSS >3 (26). Correspondingly, with regards to employment, a Norwegian study found that adjusted *OR* for employment was 0.05 (95% *CI*, 0.01–0.26) for those with EDSS > 6 when compared with those with EDSS 0–3; and *OR* for employment in Hong Kong was 0.071 (95% *CI*, 0.003–1.775) for those with EDSS >5.5 (23).

To sum up, all identified studies reported higher unemployment, higher early retirement, and higher odds for unemployment in relation to higher physical disability.

Additionally, we identified three studies investigating the employment-related outcomes within different levels of cognitive function. A study in the United Kingdom (25) reported higher employment rate in relation to higher SDMT scores, e.g., 100% with SDMT 60–80, 60% with SDMT 50–60, 45% with SDMT 40–50, 20% with SDMT 30–40, and 0% with SDMT 20–30, concluding that SDMT was the most significant predictor of unemployment. Similarly, two studies in the United States explored odds for unemployment associated with changes in SDMT: Morrow et al. (27) concluded that decline on neuropsychological tests, such as SDMT, over time is predictive of deterioration in vocational status, and Fraser et al. (28), summarizing that relatively brief, simple tests (such as SDMT) appear to be very tangible predictors of one's ability to both secure and retain employment.

### Income-Related Outcomes

In total, five cross-sectional studies investigated income-related outcomes; four of them with regards to physical disability (Table 4), and one—with regards to cognitive function (Table 5). The largest study in Sweden (9) that included 7,929 patients with MS comprehensively described income of persons with MS in relation to physical disability level, and found significant correlations between greater disability and lower earnings and higher income from benefits: individuals with severe disability had 59% lower earnings and 92% higher benefits than patients with mild disability. In addition, the proportion of patients receiving some type of benefits was two times as high in the group with severe disability—where almost everyone received benefits—compared with the group of patients with mild disability. The patients with MS with severe disability (EDSS ≥ 7) had on average SEK 166,931 less annual income from earnings and SEK 54,534 more income from benefits (~EUR 17,600 and EUR 5,700, respectively) compared to those with mild disability. Persons with MS with mild and moderate mild disability (EDSS 0–5.5), mostly had earnings, whereas those with moderate severe and severe disability (EDSS 6–9.5) had their main source of income from disability pension. The adjusted risk ratio for having earnings among persons with MS with severe disability compared with the persons with mild disability was 0.33 (95% *CI*, 0.29–0.39), while the risk ratio for receiving benefits was 1.93 (95% *CI*, 1.90–1.94).

Similarly, the other two studies reported an increasing proportion of persons with MS on benefits with increasing physical disability: from 40% receiving disability related income at EDSS 0–4.5 to 91% at EDSS 7–9.5 in the United Kingdom (24) and from 1.7% receiving invalidity pension at EDSS 0–3 to 39.1% at EDSS 7–9 in Italy (19). In New Zealand, the median annual income for those with greater disability was two times lower (NZD 15,000 at EDSS > 6 and NZD 30,000 at EDSS <3) (18).

To summarize, the studies pointed out significant correlations between greater disability and lower earnings and higher income from benefits.

With regards to the cognitive function and income among persons with MS, a Swedish study (12) thoroughly explored this, assessed with SDMT: persons in the highest SDMT score quartile earned more than two times annually compared with those in the lowest quartile, whereas persons in the lowest quartile received three times more income through social benefits. The difference in earnings and benefits across the SDMT performance quartiles remained statistically significant after adjusting for various clinical and socio-demographic variables, such as physical disability. The corrected prevalence ratios for persons with MS in the highest quartile having income from earnings and benefits were 1.40 (95% *CI*, 1.29–1.49) and 0.81 (95% *CI*, 0.71–0.90), respectively, when compared with the persons in the lowest quartile.

### Work Ability-Related Outcomes

We identified five studies that investigated work ability-related outcomes, two of them were cohort and three were cross-sectional in study design. Two studies explored work ability in relation to disability (Table 6) (29, 32), one—in relation to cognitive function (Table 7) (30), and two in relation to both disability and cognition (Tables 6, 7) (31, 33). Two Swedish studies assessed the risk for work ability: Kavaliunas et al. (33) reported increasing adjusted incidence rate ratios (IRRs) with higher disability both at 1- and 3-year follow-ups: 2.42 (95% *CI*, 1.72–3.39) and 2.61 (95% *CI*, 1.72–3.39), respectively, at EDSS 7.9–5 when compared with those with EDSS 0–3.5. Whereas, Sundstrom et al. (29) reported both increasing odds for full sick leave and partial or full sick leave with greater disability: 34 (95% *CI*, 13–86) and 150 (95% *CI*, 19–1,200), respectively, at EDSS >6 when compared with those with EDSS 0–3.5. Another study in the United States (31) investigated disability in relation to absenteeism (missing work because of health problems) and presenteeism (impairment while working) and concluded that statistically significant correlations (0.21–0.43) were found between presenteeism (but not absenteeism) and increasing disability.

With regards to cognitive function, Kavaliunas et al. (33) reported that after 1 year of follow-up, those in the lowest SDMT quartile were estimated to have a 73% higher rate of work disability (operationalized as annual net days of sickness absence and/or disability pension) when compared with those in the highest SDMT quartile (IRR 1.73; 95% *CI*, 1.42–2.10). At 3-year follow-up this estimate was similar (IRR = 1.68; 95% *CI*, 1.40–2.02). In addition, another Swedish study (30) pointed to a higher proportion not on full-time disability pension among those without cognitive impairment when compared with those with cognitive impairment (57% and 43%, respectively). However, previously mentioned study in the United States (31) did not find significant correlations between absenteeism/presenteeism and cognitive function.

To sum up, the studies reported higher work disability in relation to higher physical disability and lower cognitive function.

### Relationship Outcomes

There were two studies conducted in Sweden that reported relationship status, one cross-sectional (9) and one cohort study (Tables 8, 9) (33). None of the study investigated the relationship status as a socioeconomic outcome but reported only the respective proportions. With regards to physical disability, the proportion of the most common family composition—living with a partner and with children—decreased with greater disability from 44.1% at EDSS 0–3.5 to 17.3% at EDSS 7–9.5; the most common family composition among the most disabled patients (EDSS 7–9.5) was to live alone (single and without children—52.7%) (9). With regards to cognitive function, family composition did not differ significantly across SDMT quartiles (*p* > 0.05) (33).

### Education Level

Information on the formal education level was available and extracted from the three included studies (Tables 8, 9) out of 19. Secondary education was the most common educational level among the persons with MS, overall (46.8%) and in the different disability groups, while the percentage of persons with lower education increased from 8.2% to 18.6% and the percentage of persons with higher education decreased from 46.5% to 31.6% with greater disability (9).

As expected, when comparing persons with MS with the highest cognitive function in the fourth quartile (QIV) to those with the lowest cognitive function in the first quartile (QI), a smaller proportion had a lower educational level: 2.5 vs. 15.8%, respectively (12); and 42.4 vs. 65.2%, respectively, for lower or secondary education (33).

## Discussion

In this systematic review of the socioeconomic consequences of MS, we summarized findings of differences between persons with MS with regards to the physical disability and cognitive function in terms of employment, income, work ability, family status, and education. All identified studies reported higher unemployment, higher early retirement, and higher odds for unemployment in relation to higher physical disability. In addition, cognitive function was found to be a predictor of employment (unemployment). The studies pointed out significant correlations between greater disability and lower earnings and higher income from benefits. Besides, one identified study showed the similar results with regard to the cognitive functions. The studies reported higher work disability in relation to higher physical disability and lower cognitive function.

Our results are in line with other studies that reported similar findings with regards to physical disability. For example, Findling et al. (22) reported median EDSS among fulltime working, part-time working, and among fulltime retired, which were 2.0, 3.0, and 5.0, respectively. Similarly, Koziarska et al. (26) reported a higher mean EDSS score among unemployed when compared with employed, 3.18 and 1.57, respectively. Corresponding results were also presented by Cadden et al. (34) (mean EDSS score among unemployed was 5.0 vs. 3.8 among employed), and Strober et al. (35) (4.62 and 3.13, respectively), as well as by Strober et al. (36) among women with MS (5.52 and 4.05, respectively). Lode et al. (37) reported mean EDSS score among those on disability pension—4.0 and among those not on disability pension—2.4.

In addition, Campbell et al. (25) and Lau et al. (23) reported mean SDMT among employed and unemployed: 53.3 vs. 39.5 and 50.73 vs. 33.35, respectively. Similar results, showing higher SDMT means among employed when compared with unemployed, were reported in several studies by Strober et al.: 43.69 vs. 34.28, respectively (35), and 57.03 vs. 48.03, respectively (38), or 53.59 vs. 45.52, respectively (36). Additionally, Goverover et al. (39) found that the SDMT score among those employed and able to cook was 57.4, among those unemployed but able to cook-−48.2, among employed who did not cook—42.6, and among unemployed who did not cook—44.5. Lode et al. (37) reported mean SDMT score among those on disability pension—40.0 and among those not on disability pension—49.7. Furthermore, Moore et al. (40) using a multinomial logistic regression revealed the factors most strongly predictive of employment status were disability level, years of education, disease duration, and fatigue.

This is in line with a study that showed employment status to be associated with the Processing Speed Test (adaptation of SDMT) (41), whereas EDSS and SDMT were among the strongest predictors of employment status (35). In another study, higher level of disability and lower level of education at baseline predicted loss of employment at follow-up, however, not cognitive function (self-reported) (42).

Among the strengths of our study is that we assessed a wide spectrum of socioeconomic outcomes. As listed in Table 1, it is a heterogeneous field of investigation. Our mapping of these outcomes could help to define study outcomes when designing a study aiming at more comparable results and outcomes. Thus, we suggest reporting ratios (e.g., prevalence ratio for having income, *OR* for sick leave, IRR for work disability) instead of proportions.

Due to this wide spectrum, it was not possible to assess the extracted information from the studies in a quantitative manner. In addition, the majority of the studies were cross-sectional in design. It is important to underscore that due to well-known limitations of cross-sectional designs, the correlations revealed in the studies may not necessarily be causative (e.g., high cognitive reserve (i.e., high employment status), may protect against cognitive decline, just as cognitive decline may contribute to unemployment in persons with MS). Given the chronic and progressive nature of MS, more studies with longitudinal approach are needed for more robust measures. Additionally, we were specifically looking into physical disability as assessed by EDSS and cognition as assessed by SDMT, however, there are many more various assessments and evaluations used in the clinical practice. Furthermore, the generalizability of the studies may be limited to countries with a similarly functioning labor market and welfare system. One additional aspect that can be explored further is comorbidity, as a study in Denmark found that both psychiatric and somatic comorbidity implied an increased risk of a low income 10 years after MS onset (43). A study in Sweden also concluded that psychiatric diagnoses and medications are common among patients with MS and adversely affect risk for disability pension (44).

By reviewing and summarizing the studies investigating the socioeconomic consequences, we illustrate how such outcomes can be used to study MS. The high correlation between EDSS and both earnings and benefits indicate that these could be used as proxies for disability in registry studies investigating factors of importance for MS progression (9). Cognitive function affects the financial situation of persons with MS negatively and independently of physical disability. This warrants cognitive testing as a routine measure at follow-ups for persons with MS. This is remarkable since SDMT by no means covers more than some aspects of cognitive impairment in MS. In addition, SDMT has outstanding qualities (superior reliability, sensitivity, greater patient acceptance, better psychometric validity and ease of administration compared with other processing speed tests, good correlation with MRI data, and with activities of daily living and employment) (45, 46). Within a brief battery of cognitive tests, the SDMT was found to be the test that best predicted future cognitive decline (47). SDMT was the only neuropsychological test which predicted impaired money management in patients with MS (48). Thus, a full cognitive assessment is likely to be more predictive of reduction of earnings. To allow persons with MS to adapt optimally to their situation, mapping of cognitive function should be considered mandatory in healthcare services (12). Cognitive function is, to a high extent, associated with future work disability in persons with MS, after adjusting for other factors. An interesting aspect that has also arisen from the results is the possible association of EDSS and SDMT—as patients with MS in the highest SDMT quartile had lower EDSS scores, i.e., a median of 2.0, whereas the patients in the lowest SDMT quartile had the median EDSS score of 4.0. Whether these measures are of different construct or reflect disease progression in a similar way, as well as how they change through the clinical course in relation to each other, might be well explored in future studies (33).

In conclusion, this systematic review summarizes the pronounced differences in various socioeconomic outcomes between persons with MS in regard to their physical disability and cognitive function. In addition, we identified lack of studies with longitudinal design in this field that can provide more robust estimates with covariate adjustments, such as the disease modifying treatments.

## Data Availability Statement

The original contributions presented in the study are included in the article/Supplementary Material, further inquiries can be directed to the corresponding author.

## Author Contributions

AK and JH conceived and planned the study. AK, VD, and JH defined the search strategy. AK conducted the first screening of potentially relevant records based on titles and abstracts. AK and VD independently performed the final selection of included studies based on full text evaluation. AK, VD, and SB independently assessed the quality of the included studies. AK drafted the manuscript. VD, SB, and JH reviewed and edited the manuscript. All authors contributed to the article and approved the submitted version.

## Funding

The study was financially supported by Biogen. Biogen courtesy reviewed the manuscript and provided feedback to the authors. The authors had full editorial control and provided approval to final content.

## Conflict of Interest

AK is also employed by Takeda Pharma AB. VD has received financial support from Stockholm County Council; Biogen (recipient of grant and scholarship, PI for project sponsored by); Novartis (Scientific Advisory board member, recipient of scholarship and lecture honoraria); Merc (Scientific Advisory Board member, recipient of lecture honoraria). SB has received speaker's fee and had travel and conference expenses paid by Biogen. JH received honoraria for serving on advisory boards for Biogen and Novartis and speakers fees from Biogen, MerckSerono, BayerSchering, Teva, and SanofiGenzyme. He has served as P.I. for projects sponsored by, or received unrestricted research support from Biogen, SanofiGenzyme, MerckSerono, TEVA, Novartis, and BayerSchering. His MS research is funded by the Swedish Research Council and the Swedish Brain Foundation. The authors declare that none of the funders were involved in the study design, collection, analysis, interpretation of data, the writing of this article, or the decision to submit it for publication.

## Publisher's Note

All claims expressed in this article are solely those of the authors and do not necessarily represent those of their affiliated organizations, or those of the publisher, the editors and the reviewers. Any product that may be evaluated in this article, or claim that may be made by its manufacturer, is not guaranteed or endorsed by the publisher.
