# Supplementary material for: Systematic Review of the Socioeconomic Consequences in Patients With Multiple Sclerosis With Different Levels of Disability and Cognitive Function
Source: Front Neurol. 2022 Jan 6;12:737211. doi: 10.3389/fneur.2021.737211 (PMC8770980; doi:10.3389/fneur.2021.737211)
Supplement: Supplementary file 1 [file Table_1.DOCX]

**Supplementary Material 1. Search strategy**

**1. Medline**

| Interface: Ovid  Date of Search: 1 July 2019  Number of hits: 2,345  Comment: In Ovid, two or more words are automatically searched as phrases; i.e. no quotation marks are needed | Field labels   - exp/ = exploded MeSH term - / = non exploded MeSH term - .ti,ab,kf. = title, abstract and author keywords - adjx = adjacent within x words, regardless of order - * = truncation of word for alternate endings |
| --- | --- |
| 1. exp Multiple Sclerosis/ 2. multiple sclerosis.ti,ab,kf. 3. or/1-2  4. exp Socioeconomic Factors/ 5. Absenteeism/ 6. Presenteeism/ 7. exp Insurance, Disability/ 8. exp Family Relations/ 9. Social Participation/ 11. ((education* or social or socioeconomic* or socio-economic*) adj3 (class or consequence* or factor* or impact* or level or participation or position* or status)).ti,ab,kf. 12. ((economic* or financ*) adj3 (burden or characteristic* or compensation or consequence* or remuneration or situation or status or support)).ti,ab,kf. 13. ((disability or sick*) adj3 (absence* or benefit* or compensation* or cost* or day* or insurance* or leave or pay* or pension* or presence or remuneration)).ti,ab,kf. 14. ((family or marital or partner*) adj3 (characteristic* or relation* or situation* or status)).ti,ab,kf. 15. ((labor or labour or work*) adj3 (ability or disability or capacity or incapacity)).ti,ab,kf. 16. ((physical or social) adj3 function*).ti,ab,kf. 17. or/4-16  18. 3 and 17  19. limit 18 to english language | |

**2. Embase**

| Interface: embase.com  Date of Search: 1 July 2019  Number of hits: 3,749  Comment: Emtree is the controlled vocabulary in Embase | Field labels   - /exp = exploded Emtree term - /de = non exploded Emtree term - ti,ab = title and abstract - NEAR/x = adjacent within x words, regardless of order - * = truncation of word for alternate endings |
| --- | --- |
| #1 'multiple sclerosis'/mj #2 'multiple sclerosis':ti,ab,kw #3 #1 OR #2  #4 'socioeconomics'/mj #5 'employment status'/exp/mj #6 'educational status'/mj #7 'household income'/exp/mj #8 'social status'/exp/mj #9 'absenteeism'/mj #10 'presenteeism'/mj #11 'insurance'/exp/mj #12 'social participation'/mj #13 absentee*:ti,ab,kw OR career:ti,ab,kw OR divorce:ti,ab,kw OR earning*:ti,ab,kw OR employment:ti,ab,kw OR income:ti,ab,kw OR marriage:ti,ab,kw OR money:ti,ab,kw OR presentee*:ti,ab,kw OR retire*:ti,ab,kw OR salary:ti,ab,kw OR salaries:ti,ab,kw OR ses:ti,ab,kw OR spouse*:ti,ab,kw OR unemployment:ti,ab,kw OR wage*:ti,ab,kw #14 ((education* OR social OR socioeconomic* OR 'socio economic*') NEAR/3 (class OR consequence* OR factor* OR impact* OR level OR participation OR position* OR status)):ti,ab,kw #15 ((economic* OR financ*) NEAR/3 (burden OR characteristic* OR compensation OR consequence* OR remuneration OR situation OR status OR support)):ti,ab,kw #16 ((disability OR sick*) NEAR/3 (absence* OR benefit* OR compensation* OR cost* OR day* OR insurance* OR leave OR pay* OR pension* OR presence OR remuneration)):ti,ab,kw #17 ((family OR marital OR partner*) NEAR/3 (characteristic* OR relation* OR situation* OR status)):ti,ab,kw #18 ((labor OR labour OR work*) NEAR/3 (ability OR disability OR capacity OR incapacity)):ti,ab,kw #19 ((physical OR social) NEAR/3 function*):ti,ab,kw #20 #4 OR #5 OR #6 OR #7 OR #8 OR #9 OR #10 OR #11 OR #12 OR #13 OR #14 OR #15 OR #16 OR #17 OR #18 OR #19  #21 #3 AND #20 AND [english]/lim | |

**3. Web of Science Core Collection**

| Interface: Clarivate Analytics  Date of Search: 1 July 2019  Number of hits: 2,342 | Field labels   - TS/Topic = title, abstract, author keywords and Keywords Plus - NEAR/x = adjacent within x words, regardless of order - * = truncation of word for alternate endings |
| --- | --- |
| #1 TOPIC: (”multiple sclerosis”)  #2 TOPIC: ((absentee* or career or divorce or earning* or employment or income or marriage or money or presentee* or retire* or salary or salaries or SES or spouse* or unemployment or wage*)) OR TOPIC: (((education* or social or socioeconomic* or socio-economic*) NEAR/3 (class or consequence* or factor* or impact* or level or participation or position* or status))) OR TOPIC: (((economic* or financ*) NEAR/3 (burden or characteristic* or compensation or consequence* or remuneration or situation or status or support))) OR TOPIC: (((disability or sick*) NEAR/3 (absence* or benefit* or compensation* or cost* or day* or insurance* or leave or pay* or pension* or presence or remuneration))) OR TOPIC: (((family or marital or partner*) NEAR/3 (characteristic* or relation* or situation* or status))) OR TOPIC: (((labor or labour or work*) NEAR/3 (ability or disability or capacity or incapacity))) OR TOPIC: (((physical or social) adj3 function*))  #3 #1 AND #2 Timespan=1945-2019, Refined by: LANGUAGES: ( ENGLISH ) | |
