# Supplementary material for: Systematic Review of the Socioeconomic Consequences in Patients With Multiple Sclerosis With Different Levels of Disability and Cognitive Function
Source: Front Neurol. 2022 Jan 6;12:737211. doi: 10.3389/fneur.2021.737211 (PMC8770980; doi:10.3389/fneur.2021.737211)
Supplement: Supplementary file 2 [file Table_2.DOCX]

**Supplementary Material 2. Quality assessment of the selected studies**

| **#** | **First Author (year)** | **Time** | **Study design** | **Data source** | **Population** | **Grade** |
| --- | --- | --- | --- | --- | --- | --- |
|  | Battaglia (2017)^19^ | 2015 | Cross-sectional | Questionnaires | 1010 MS patients | B |
|  | Boe Lunde (2014)^20^ | 2008-2010 | Cross-sectional | Interviews  Questionnaires | 213 MS patients | C |
|  | Busche (2003)^21^ | 1996-1997 | Cross-sectional | Interviews | 96 MS patients under age 65 | C |
|  | Campbell (2016)^26^ | 2015-2015 | Cross-sectional | Questionnaires | 62 MS patients | C |
|  | Chruzander (2016)^31^ | 1994-2012 | Cohort | Registries | 114 MS patients | A |
|  | Doesburg (2019)^29^ | 2012-2015 | Cross-sectional | Questionnaires | 90 MS patients | C |
|  | Findling (2015)^22^ | 2009 | Cross-sectional | Questionnaires | 405 MS patients | C |
|  | Fraser (2009)^28^ | N/A | Cross-sectional | Interviews | 95 MS patients | C |
|  | Glanz (2012)^32^ | 2010 | Cross-sectional | Questionnaires | 377 with CIS or RRMS | C |
|  | Kavaliunas (2015)^9^ | 2010 | Cross-sectional | Registries | 7929 MS patients aged 21-64 years | B |
|  | Kavaliunas (2019)^12^ | 2010 | Cross-sectional | Registries | 2080 MS patients 21-64 years | B |
|  | Kavaliunas (2019)^33^ | 2006-2012 | Cohort | Registries | 903 MS patients aged 19-65 | A |
|  | Kobelt (2017)^17^ | 2015-2016 | Cross-sectional | Questionnaires | 16,808 MS patients | B |
|  | Koziarska (2018)^25^ | 2015-2016 | Cross-sectional | Questionnaires | 150 MS patients receiving disease modifying therapies | C |
|  | Lau (2016)^23^ | 2010-2011 | Cross-sectional | N/A | 59 MS patients | C |
|  | MacLurg (2005)^24^ | 2001 | Cross-sectional | Interviews | 149 MS patients | C |
|  | Morrow (2010)^27^ | N/A | Cohort | Questionnaires | 97 employed MS patients | C |
|  | Pearson (2017)^18^ | 2006 | Cross-sectional | Survey | 1727 MS patients 25-64 years | B |
|  | Sundström (2003)^30^ | 1997 | Cross-sectional | Interviews | 399 MS patients | C |

A – high quality; B – moderate quality; C – low quality; N/A – not available.
